# Congenital hip dysplasia treated by total hip arthroplasty using cementless tapered stem in patients younger than 50 years old: results after 12-years follow-up

**DOI:** 10.1007/s10195-011-0170-y

**Published:** 2011-11-24

**Authors:** Cesare Faldini, Maria Teresa Miscione, Mohammadreza Chehrassan, Francesco Acri, Camilla Pungetti, Michele d’Amato, Deianira Luciani, Sandro Giannini

**Affiliations:** Department of Orthopaedic Surgery, Istituto Ortopedico Rizzoli, University of Bologna, Via G. Pupilli 1, 40136 Bologna, Italy

**Keywords:** Congenital hip dysplasia, Total hip arthroplasty, Cementless tapered stem, Long-term follow-up

## Abstract

**Background:**

Congenital hip dysplasia may lead to severe acetabular and femoral abnormalities that can make total hip arthroplasty a challenging procedure. We assessed a series of patients affected by developmental hip dysplasia treated with total hip arthroplasty using cementless tapered stem and here we report the outcomes at long-term follow-up.

**Materials and methods:**

Twenty-eight patients (24 women and 4 men) aged between 44 and 50 years (mean 47 years) were observed. Clinical evaluation was rated with the Harris Hip Score. Radiographic evaluation consisted in standard anteroposterior and axial view radiographs of the hip. According to Crowe’s classification, 16 hips presented dysplasia grade 1, 14 grade 2, and 4 grade 3. All patients were treated with total hip arthroplasty using a cementless tapered stem (Wagner Cone Prosthesis). Six patients were operated bilaterally, with a totally of 34 hips operated. After surgery, the patients were clinically and radiographically checked at 3, 6, and 12 months and yearly thereafter until an average follow-up of 12 years (range 10–14 years).

**Results:**

Average Harris Hip Score was 56 ± 9 (range 45–69) preoperatively, 90 ± 9 (range 81–100) 12 months after surgery, and 91 ± 8 (range 83–100) at last follow-up. Radiographic evaluation demonstrated excellent osteointegration of the implants. Signs of bone resorption were present in 6 hips, nevertheless no evidence of loosening was observed and none of the implants has been revised.

**Conclusions:**

Even in dysplasic femur, the tapered stem allowed adequate stability and orientation of the implant. We consider tapered stem a suitable option for total hip arthroplasty in developmental hip dysplasia, also in case of young patients, thanks to the favourable long-term results.

## Introduction

**Fig. 1:**
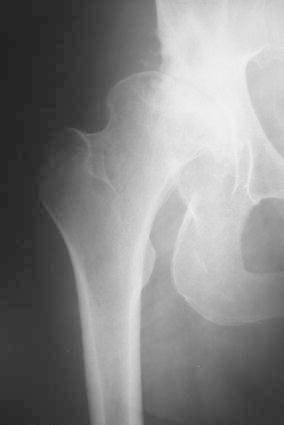
Preoperative radiographic aspect in anteroposterior view of a 59 year-old female with Crowe grade 1 congenital hip dysplasia

**Fig. 2:**
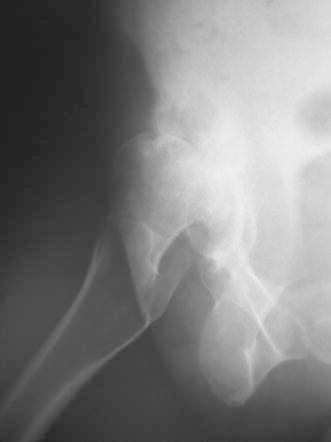
Preoperative radiographic aspect in axial view

Congenital hip dysplasia (or developmental hip dysplasia) consists of an abnormal development of the hip joint characterized by anatomical alterations involving both the acetabulum and the femur. If not diagnosed and adequately treated during childhood, congenital hip dysplasia may lead to early functional impairment in young adults: the acetabulum typically presents reduced depth with superior deficiency; the femur usually presents deformed head, short neck with excessive anteversion, small and unusually shaped medullary canal, with a smaller medio–lateral diameter respect to its antero-posterior, and more often a greater trochanter located more posteriorly [1, 2]. The consequent mismatch of the articular surfaces, associated with the shortening of the lower limb and the unbalanced abductor muscle strength, produces overtime pain, limping and finally secondary arthritis of the hip joint [2] (Figs. 1, 2).

The characteristic pathological anatomy, the early onset of symptoms and degenerative joint changes, and the relatively young age of the patients make the treatment of developmental hip dysplasia in adults a challenge for surgery [3–6]: many surgical strategies, including acetabular reconstruction, femoral osteotomies and joint arthroplasty, have been proposed over the years. Regarding total hip arthroplasty in developmental hip dysplasia, the treatment of choice is still widely debated [7–25]. The anatomical alterations make the selection of the prosthesis complex and the implant technically demanding. Moreover, previous surgery (e.g. femoral osteotomies, acetabuloplasty) can highly increase the difficulty of joint replacement. Finally, the relatively young age of the patients highlights the problem of the long-term survival of the implants [8, 11–15].

Most part of literature suggests to place the acetabular cup in the anatomical position in order to equalize lower limb length and to achieve adequate abductor muscle strength [7, 21, 22, 26]. However, this procedure requires an adequate coverage of the acetabulum, otherwise reconstruction of the superior deficiency or medialization of a smaller prosthetic component is necessary [16, 22, 26, 27]. Many authors report longer duration of uncemented implants compared to cemented ones, and a simpler revision surgery in case of implant loosening; thus, the use of cementless stems in young patients is suggested [8, 14, 15, 20, 24, 25]. Anyway, the implant of anatomical cementless stems appears difficult in case of severe femoral deformity, exposing the patient to greater risks of intraoperative fractures and incorrect placement of the implant [28, 29]. Moreover, the anteversion of the implanted acetabular component influences how much anteversion can be accepted on the femoral side without compromising hip stability [19, 27]. We believe that these problems could be avoided using tapered stems, which enable the use of a femoral cementless implant even in case of developmental hip dysplasia. The aim of this study is to review the results of a series of patients younger then 50 years old affected by developmental hip dysplasia treated by total hip arthroplasty using a cementless tapered stem, at long-term follow-up.

## Materials and methods

Forty-six patients affected by developmental hip dysplasia were treated between 1995 and 1998 with total hip arthroplasty. Inclusion criteria were mono- or bilateral congenital hip dysplasia, with pain. Exclusion criteria were any previous surgery and patients older than 50 years old. Eighteen patients had been previously treated by femoral osteotomy, so they were excluded. Twenty-eight patients (24 females and 4 males) were included in the study. Six of them underwent surgery bilaterally, for a total of 34 hips observed. Average age at time of surgery was 47 years (range 44–50 years). This study conforms to the Declaration of Helsinki as revised in 2008 and was authorized by the ethical committee of authors’ institution. All patients gave the informed consent to this study.

Preoperatively, all patients were evaluated clinically and radiographically. For each patient, the complete medical history was collected and pain and grade of disability were assessed in terms of limitation of the hip range of motion, limb length discrepancy, and restrictions on walking and daily activities. Clinical evaluation was rated with the Harris Hip Score [31]. For all patients, standard radiographs of the pelvis in anteroposterior view and of the affected hip in axial view were obtained (Figs. 1, 2). On radiographs, the degree of dysplasia was determined using the system of Crowe [32], and according to this, 16 hips presented dysplasia grade 1, 14 grade 2, and 4 grade 3. The transverse diameter of the medullary canal and the thickness of the cortex of the femur were also assessed. The appropriate implant was chosen by preoperative templating.

Surgery was performed under general or spinal anaesthesia. In all cases, postero-lateral approach was used, with the patient placed on the contralateral side. One cm posteriorly from the tip of the greater trochanter, a skin incision, extended straight for approximately 15 cm, was performed. The subcutaneous tissue and the gluteal fascia were incised and retracted medially together with the gluteus maximus. The gluteus medius was retracted and the short extrarotators were isolated and detached. The insertion of the quadratus was spared if possible. Capsulotomy was performed, and the hip was dislocated by pulling, flexing, and internally rotating the lower limb. The femoral neck was resected at its base and the femoral head was removed.

The primitive acetabulum was reamed, and a small, uncemented, press-fit cup was applied in or close to the anatomical position. Metal-polyethylene coupling was used in 27 cases, while in 7 cases ceramic–ceramic coupling was used.

Since the dysplastic femur presents abnormal neck anteversion, in this series we did not consider the femoral neck to be a suitable reference for stem placement, therefore the correct orientation of the stem was determined referring to the condylar plane. With the hip 45° flexed and 90° internally rotated and the knee 90° flexed, the condylar plane is almost perpendicular to the longitudinal tibial axis: anteversion was determined by using the tibial axis as a reference point. A Steinmann pin was inserted into the tip of the greater trochanter, parallel to the longitudinal tibial axis.

The medullary canal was reamed using tapered reamers of progressively increasing size until the planned size was achieved and until resistance of the reamer against the femur cortex was felt. The depth of the reamer was determined by the Steinmann pin, which should correspond to the notch on the reamer. Then a Wagner Cone Prosthesis uncemented stem was inserted by guiding it with its appropriate device, aiming to obtain about 10–20° of anteversion.

After the hip has been reduced, stability and range of motion were assessed, and limb length checked in comparison to the contralateral limb.

A drainage was applied, the extrarotators were reinserted, and the gluteal fascia, the subcutaneous tissue, and the skin were sutured.

**Fig. 3:**
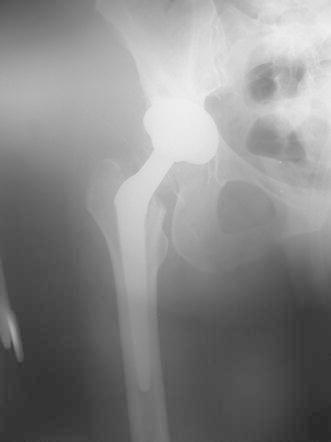
Radiographic aspect at 1 year follow-up from total hip arthroplasty showing correct placement and good integration of the implant

**Fig. 4:**
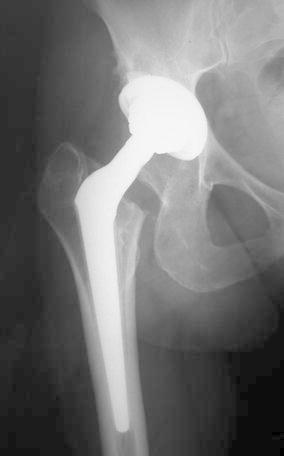
Radiographic aspect in anteroposterior view at 11 years follow-up: the implant is stable and no signs of bone resorption are noticeable

**Fig. 5:**
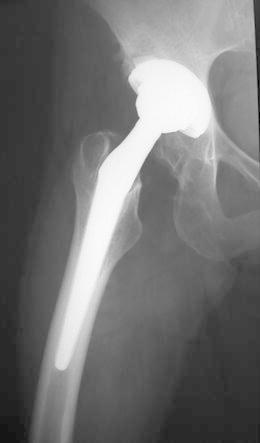
Radiographic aspect in axial view

The first day of postoperative care, the drainage was removed and physiotherapy was begun. The physical therapy was aided by a physiotherapist to make passive and active movements of the operated limb, to stand up and walk with the use of a walking frame. Initially a dragged load on the operated limb was allowed, then partial weight-bearing was allowed with the use of two crutches. All patients were discharged from 6 to 9 days (mean 7 days) after surgery, and then they were checked in the outpatient department after 1 month, when they were invited to dismiss one of the two crutches. Afterwards, all patients were checked at 2 months from surgery, when they were asked to progressively abandon the other crutch and total weight-bearing was allowed. The patients were checked clinically and radiographically 3, 6 and 12 months after surgery (Fig. 3) and yearly thereafter until an average follow-up of 12 years (range 10–14 years) (Figs. 4, 5). Twelve months after surgery and at last available follow-up, the clinical outcome was rated using the Harris Hip Score. On radiographs, the presence of osteolysis, defined as radiolucent lines surrounding the stem (according to Gruen [33]) was carefully evaluated. Moreover, stress shielding (as described by Engh [34]) was assessed.

All continuous data were expressed as mean and standard deviation of the mean, and the paired *T* test was performed to compare preoperative and postoperative scores; *p* < 0.05 was considered significant.

## Results

One intraoperative complication was reported: one intraoperative proximal femur fracture occurred, involving the greater trochanter, and treated by cerclage wiring and dragged load on the operated limb the first 5 weeks after surgery. No early infection or wound healing problems occurred. One patient underwent a deep venous thrombosis that was adequately managed on postoperative care and resolved without sequelae. One dislocation occurred, treated with close reduction and a hip brace, without the need for further surgery, and no recurrence was observed. No septic or aseptic loosening of the implants was observed.

Average Harris Hip Score was 56 ± 9 (range 45–69) preoperatively, 90 ± 9 (range 81–100) at 12 months after surgery, and 91 ± 8 (range 83–100) at last follow-up.

All patients showed clinical improvement after surgery. All patients walked normally without any help at 3 months after surgery. Only 2 patients with a limb length discrepancy of about 2 cm required the use of a shoe lift; however, it was well tolerated. This device satisfactorily solved the problem and allowed the patients to walk normally without limping. All patients declared that they were satisfied with the results of surgery in terms of hip function and relief from pain.

Radiographic evaluation demonstrated excellent osteointegration of the implants in most cases.

At last follow-up, bone resorption and stress shielding were noticeable in 6 hips (grade 1 in 4 hips, grade 2 in 2 hips, as described by Engh [34]), nevertheless in these cases no definite clinical or radiographic evidence of implant loosening was observed. None of the implants has been revised. Clinic, limb length discrepancy and radiographic resorption were not related to the grade of dysplasia.

## Discussion

Congenital hip dysplasia can lead to severe anatomical alterations of the hip joint. These alterations increase the difficulty of joint replacement, and may also threaten the long-term survival of the prosthetic implant. Appropriate selection of the prosthesis and a carefully conducted surgical procedure are thus essential to ensure the best result and the longevity of the implant. Total hip arthroplasty using a cementless tapered stem demonstrated to be effective for the treatment of developmental hip dysplasia, allowing relief from symptoms and improvement of hip function, with durable outcomes at long-term follow-up [30].

The disadvantages of cementless anatomical stems are reduced thanks to the characteristics of the tapered stem, allowing to obtain adequate stability and good orientation of the implant, even in a displastic femur. In our series, radiographic evaluation at follow-up showed in most cases osteointegration of the stem in the proximal region of the femur. We believe this phenomenon is due to the conical geometry of the prosthesis. In fact, in the tapered stem, the surface per unit of length increases with the diameter, and the surface area is greater in the proximal part of the stem than in the distal part. With a continuous surface contact between the stem and the bony interface, a uniform load transfer per unit of area can be assumed; therefore, since the load is distributed per surface unit, in a conical prosthesis the load is greater in the proximal region compared to the distal one. We believe that this feature may prevent stress-shielding and load by-pass in a proximal–distal direction, avoiding bone resorption of the proximal portion of the femur with subsequent secondary instability of the implant, even if the role of stress-shielding (and particularly its influence on clinical outcome) is still not clearly demonstrated [20, 34–42]. Besides, by avoiding greater and abnormal loads on the distal portion of the stem and the resulting pedestal formation, the onset of thigh pain is prevented; which conversely is reported after cementless total hip arthroplasty, especially in patients treated with long prosthetic stems [23, 43, 44]. We are also aware that the tapered prosthesis should not be used in cylindrical femurs with thin cortices, because we believe it would be inappropriate to weaken the cortex by conically reaming the canal.

In case of anatomical stems with prismatic cross-sections, the altered shape of the medullary canal could force the implant to rotate, and the surgeon might be unable to control the anteversion angle [45]. This feature accounts for the higher rate of intraoperative fracture and incorrect placement and fitting reported with the use of cementless anatomical stems, which sometimes lead to implant an undersized prosthesis [22, 23, 28, 29]. On the other hand, the position of the tapered stem with regards to rotation (anteversion or retroversion) is not determined by the anatomical characteristics of the dysplastic femoral epiphysis: the circular section of the tapered stem, provided with 8 longitudinal sharp ribs, allows the surgeon to choose the degree of anteversion that he considers as better.

The stem used in this series presented a fixed neck–diaphysis angle of 135°, which is rather similar to the coxa valga angle. However, in some cases, this was unfavourable for reconstructing the correct offset in hips with a reduced neck shaft angle. Therefore, we believe that it would be useful to have a range of stems with more varus deviation to be able to place the gluteal muscles under adequate tension without lengthening the limb.

In conclusion, by the using of tapered cementless stems, it is possible to achieve adequate press-fit and correct placement of the implant, especially in terms of anteversion, even in case of severe anatomical anomalies. This makes the tapered stem a suitable option for total hip arthroplasty in congenital hip dysplasia, also in case of young patients, with a low grade of complications and an optimal implant survival rate at long-term.
